# The RNA-binding proteomes from yeast to man harbour conserved enigmRBPs

**DOI:** 10.1038/ncomms10127

**Published:** 2015-12-03

**Authors:** Benedikt M. Beckmann, Rastislav Horos, Bernd Fischer, Alfredo Castello, Katrin Eichelbaum, Anne-Marie Alleaume, Thomas Schwarzl, Tomaž Curk, Sophia Foehr, Wolfgang Huber, Jeroen Krijgsveld, Matthias W. Hentze

**Affiliations:** 1 European Molecular Biology Laboratory (EMBL), Meyerhofstrasse 1, 69117 Heidelberg, Germany

## Abstract

RNA-binding proteins (RBPs) exert a broad range of biological functions. To explore the scope of RBPs across eukaryotic evolution, we determined the *in vivo* RBP repertoire of the yeast *Saccharomyces cerevisiae* and identified 678 RBPs from yeast and additionally 729 RBPs from human hepatocytic HuH-7 cells. Combined analyses of these and recently published data sets define the core RBP repertoire conserved from yeast to man. Conserved RBPs harbour defined repetitive motifs within disordered regions, which display striking evolutionary expansion. Only 60% of yeast and 73% of the human RBPs have functions assigned to RNA biology or structural motifs known to convey RNA binding, and many intensively studied proteins surprisingly emerge as RBPs (termed ‘enigmRBPs'), including almost all glycolytic enzymes, pointing to emerging connections between gene regulation and metabolism. Analyses of the mitochondrial hydroxysteroid dehydrogenase (HSD17B10) uncover the RNA-binding specificity of an enigmRBP.

## Abstract (web summary)

RNA-binding proteins (RBPs) are implicated in many biological functions. Here the authors expand the human and yeast RNA interactome identifying new and conserved RBPs, several of which with no prior function assigned to RNA biology or structural motifs known to mediate RNA-binding, and suggesting new roles of RNA as modulators of protein function.

## Graphical abstract

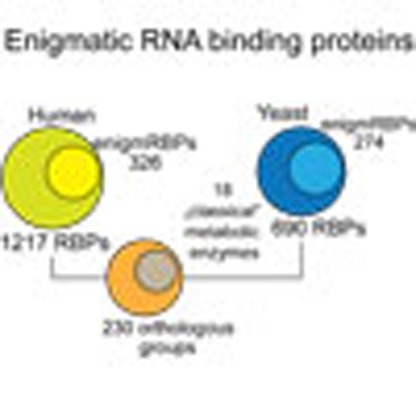

RNA-binding proteins (RBPs) mediate pivotal cellular functions such as RNA transport, degradation or translation and represent key effectors of post-transcriptional gene regulation. To fulfil such diverse roles, RBPs vary regarding their RNA-binding modes and specificities^1^. Recently developed unbiased high content techniques to identify RBPs *in vivo* yielded information on differences in cell type-specific expression and/or RNA-binding activity of RBPs in mammalian cells^2^,^3^. Apart from RBPs with defined functions^4^ in RNA biology, many other proteins, among them metabolic enzymes, have been found to bind RNA *in vivo*^5^. The recent discovery of such unorthodox RBPs using mRNA interactome capture^2^,^3^,^6^ raises the question of the evolutionary conservation and the RNA-binding specificity of such RBPs. To answer the first question, we determined the mRNA interactomes of the yeast *Saccharomyces. cerevisiae* (*S. cerevisiae*) (BY4741) and of human hepatocytic cells (HuH-7). To address the second, we investigated RNAs bound by the metabolic enzyme hydroxysteroid dehydrogenase 17-β 10 (HSD17B10). Here we identify a large set of RBPs that are conserved between yeast and human cells. We show that this conserved RNA interactome harbours many proteins without previously assigned roles in RNA biology (enigmRBPs), including surprisingly many metabolic enzymes. We also determined the RNA targets of an RNA-binding mitochondrial enzyme and show its specificity in RNA binding.

## Results

### The mRNA interactomes of yeast and human HuH-7 cells

**Figure 1:**
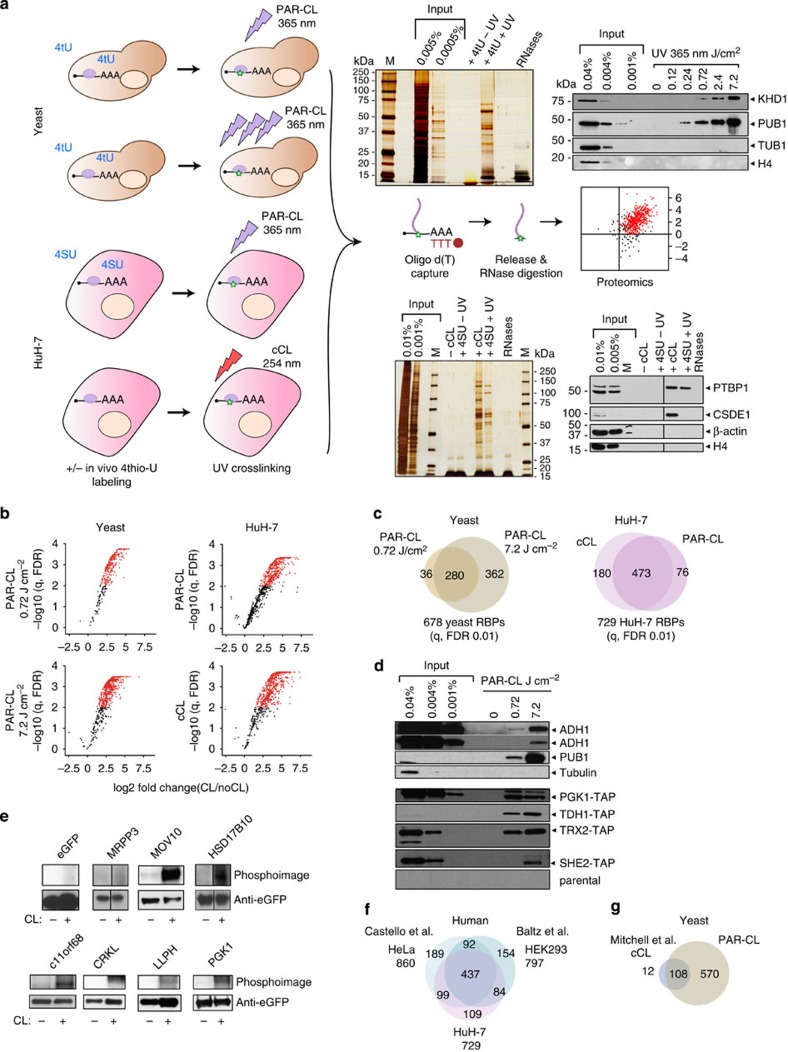
mRNA interactome capture in yeast and HuH-7 cells. (**a**) Schematic representation of the mRNA interactome capture protocol in yeast and HuH-7 cells using PAR crosslinking (PAR-CL) or conventional crosslinking (cCL). Quality controls for both yeast and HuH-7 include silver staining (middle upper and lower panel) and western blotting (right upper and lower panel) (M, protein marker, KHD1, KH-domain containing protein1 fused to eGFP, PUB1, polyU binding protein 1, TUB1, tubulin 1, H4, histone 4, PTBP1, polypyrimidine tract binding protein 1, CSDE1, cold shock domain containing protein E1). (**b**) Volcano plot showing the distribution of proteins according to their enrichment in crosslinked (CL) over non-CL samples. Proteins shown in red (FDR 0.01) represent the mRNA interactome. (**c**) Overlap of mRNA interactome proteins in yeast and HuH-7. (**d**) Validation of the yeast mRNA interactome using western blotting of input samples and eluate after interactome capture with specific antibodies (ADH1, alcohol dehydrogenase 1, PUB1) or against TAP-tagged proteins (PGK1, phosphoglycerate kinase 1, TDH1, triose phosphate dehydrogenase, TRX2, thioredoxine 2, SHE2, Swi5p-dependent HO Expression 2). (**e**) Validation of HuH-7 mRNA interactome RBPs using polynucleotide kinase-mediated ^32^P labelling after IP with an anti-eGFP antibody (negative control eGFP and MRPP3, mitochondrial RNAseP protein 3; positive control MOV10, moloney leukaemia virus 10; and novel RBPs, HSD17B10, hydroxysteroid dehydrogenase 17B 10, c11orf68, chromosome 11 open reading frame 68, CRKL, v-crk avian sarcoma virus CT10 oncogene homologue-like, LLPH, long-term synaptic facilitation homolog, PGK1, phosphoglycerate kinase 1). (**f**) Comparison of mRNA interactome RBPs from HuH-7 with those from HeLa and HEK293 cells. (**g**) Comparison of the yeast mRNA interactome RBPs to a published study using cCL^9^.

HuH-7 liver cells were exposed to ultraviolet light of 254 nm (conventional crosslinking, cCL), or of 365 nm (photo-activatable crosslinking, PAR-CL) after incubation with 4-thio-uridine^7^. We also adapted the mRNA interactome capture protocol to yeast (see Methods and Supplementary Fig. 1) using PAR-CL at 0.72 or 7.2 J cm^−2^ (ref. 8) with 4-thio-uracil. After cell lysis, polyadenylated RNAs were captured on oligo d(T) beads followed by stringent washes to remove non-crosslinked proteins. The cCL and PAR-CL samples, along with non-crosslinked controls (noCL and analogs only, respectively), were analysed by LC-MS/MS^2^ (Fig. 1a).

The yeast RBPs, KHD1 and PUB1, serving as positive controls, show dose-dependent enrichment, whereas highly abundant cellular proteins (tubulin, histones) used as specificity controls are negative (Fig. 1a, upper right panel). Similarly, the established mammalian RBPs PTBP1 and CSDE1 are specifically enriched in eluates from crosslinked HuH-7 samples (Fig. 1a, lower right). Using three biological replicates and applying stringent statistical tests (see Methods), we identify 678 (yeast) and 729 (HuH-7) high-confidence RBPs (false discovery rate (FDR) 0.01) (Fig. 1b,c; Supplementary Fig. 2; Supplementary Data 1 and 2). An additional 135 candidate RBPs are detected from HuH-7 cells at FDR 0.05 (Supplementary Data 2).

Validation experiments corroborate the quality of the data sets (Fig. 1d,e). Comparison of the HuH-7 RBPs with the HeLa and HEK293 data sets begins to define a ‘housekeeping' human mRNA interactome, while 109 HuH-7 RBPs were previously not found in other human cell types (Fig. 1f; Supplementary Data 2). The latter may be explained by cell-specific expression or culture condition-dependent RNA binding of RBPs. Our data confirm 108 of the 120 yeast RBP candidates from a recent study^9^. Importantly, we identify 570 additional yeast proteins as high-confidence RBPs (Fig. 1g; Supplementary Data 1), and an astounding >10% of the total yeast proteome thus emerge as RBPs^10^ (see below).

### Definition of the conserved eukaryotic mRNA interactome

**Figure 2:**
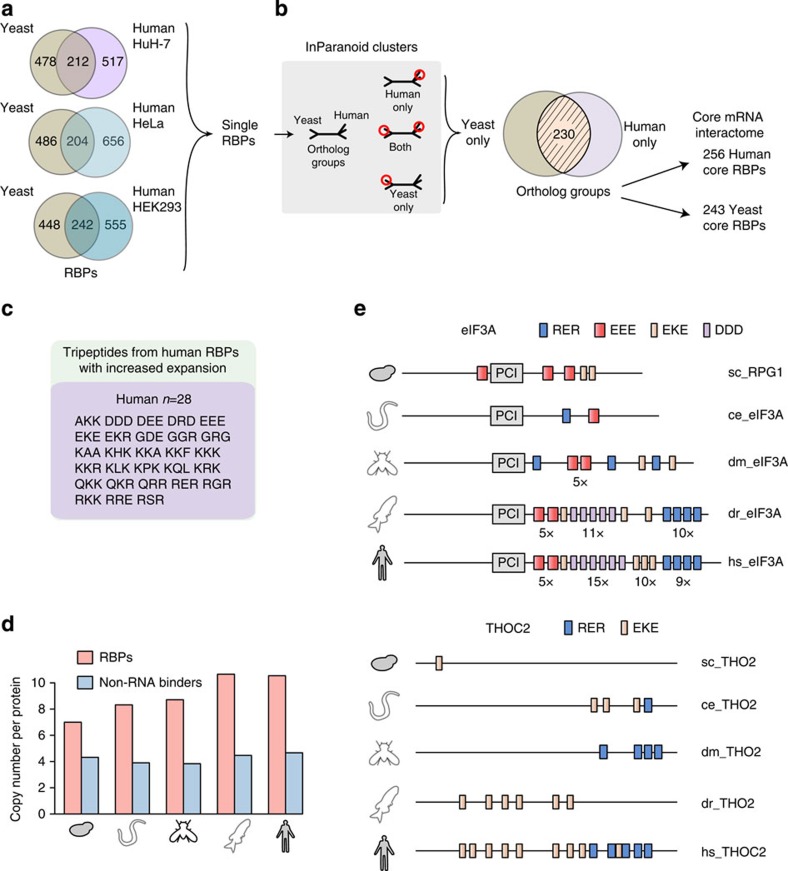
Evolutionary expansion of RBP short peptide motifs. (**a**) Overlap of yeast and human mRNA interactome orthologs. (**b**) Definition of InParanoid ortholog groups of mRNA interactome RBPs. (**c**) Tripeptide motifs showing expansion in number between human RBPs compared with their orthologous yeast RBPs (FDR 0.2). (**d**) Average copy number of motifs from (**c**), calculated for yeast, *C. elegans*, *D. melanogaster*, *D. rerio* and human orthologous RBPs of the core mRNA interactome and for non-RNA-binding orthologous proteins (non-RNA binders). (**e**) Examples of orthologous RBPs with increased copy number of motifs from **c**. Protein sequences are not drawn to scale; the relative position of domains and motifs within proteins is retained.

These sets of yeast and human RBPs were analysed for RBP conservation. Comparison of yeast with each of the three human cell lines consistently yields an overlap of >200 orthologous RBPs (Fig. 2a). We used the InParanoid database that assigns RBPs to ‘ortholog groups' of corresponding biological functions (Methods). Two-hundred and thirty ortholog groups consisting of 243 individual RBPs in yeast and 256 RBPs in human (Fig. 2b; Supplementary Data 1 and 2) constitute the conserved eukaryotic ‘core mRNA interactome'. As expected, it includes many well-studied RBPs with established functions in RNA biology and/or with well-defined RNA-binding domains (Supplementary Fig. 3a,b). Interestingly, some ‘core RBPs' share lysine [K]- and arginine [R]-rich tripeptide repeat motifs that numerically increase from yeast to human (Fig. 2c,d). Whereas their general occurrence is conserved, their number within orthologous RBPs expands with increasing complexity of the organisms. These expansions may directly interact with RNA and represent an emerging structural property of nucleic acid interactors^11^,^12^,^13^, possibly to enhance RNA-binding specificity within increasingly complex transcriptomes. The RBPs eIF3a and THOC2 are depicted as examples in Fig. 2e.

### Well-studied proteins emerge as conserved enigmRBPs

**Figure 3:**
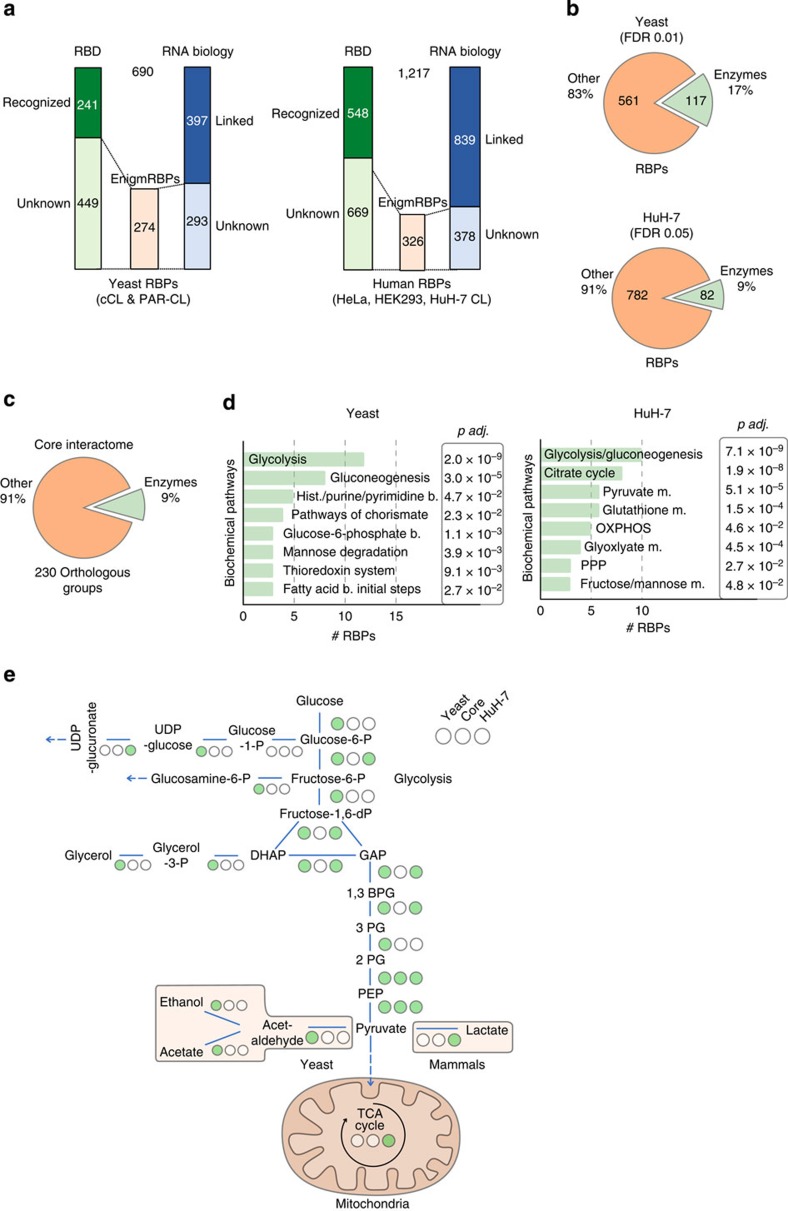
Yeast and human mRNA interactomes harbour hundreds of enigmRBPs. (**a**) Yeast and human mRNA interactome protein annotations according to domains and functional characteristics. (**b**) Subset of classic metabolic enzymes within mRNA interactomes. (**c**) Proportion of classic metabolic enzymes within the conserved core mRNA interactome. (**d**) GO biochemical pathway enrichment of the enzyme-RBPs from yeast and HuH-7. (**e**) Simplified scheme of central carbon metabolism in yeast and human. Blue lines represent enzymes, filled green circles indicate activity of enzyme as RBPs, empty circles indicate lack of evidence for RNA binding.

Merging our data sets with published information on yeast^9^ (690 RBPs in total, Supplementary Data 1) and human RBPs^2^,^3^ (1,217 RBPs in total, Supplementary Table 2), we were surprised to find that 40% (274 yeast proteins) and 27% (326 human proteins), respectively, of the identified RBPs lack both recognizable RBDs and known functions in RNA biology (selection criteria see Methods). These RBPs include many well-studied proteins whose roles in RNA biology remain to be defined (Fig. 3a); we therefore termed these proteins ‘enigmRBPs'. Phosphoglycerate kinase and thioredoxin^14^,^15^ represent enigmRBPs that we directly validated for RNA binding both in human and yeast cells (Fig. 1d,e and Castello *et al*., submitted). enigmRBPs cover a wide spectrum of biological functions, including, for example, actin-binding/remodelling, protein folding, ATP-binding and enzymatic functions in classic metabolic pathways (Supplementary Table 1). enigmRBPs resemble orthodox RBPs in terms of typical features (Supplementary Fig. 3c,d) in both yeast and human cells, and constitute a surprisingly large fraction of the conserved core RBPs (42 (17%) yeast and 28 (11%) human RBPs). A few enzymes of classical biochemical pathways that moonlight as RBPs have previously been identified^16^,^17^,^18^. We thus searched the complete yeast and human RBP data sets (including the HuH-7 candidate RBPs) for ‘classic' metabolic enzymes (hereafter referred to as enzymes; for selection criteria see Methods). Yeast RBPs (17%) and 9% of the human RBPs belong to this group (Fig. 3b; Supplementary Data 1 and 2); transferases and oxidoreductases constitute more than half of these (Supplementary Fig. 3e). Strikingly, 9% of the conserved core RBPs are metabolic enzymes (Fig. 3c; Supplementary Fig. 3f), and central carbon metabolism, especially glycolysis, emerges as a hotspot for RNA-binding enzymes (Fig. 3d,e).

### RNA binding of a mitochondrial enzyme

**Figure 4:**
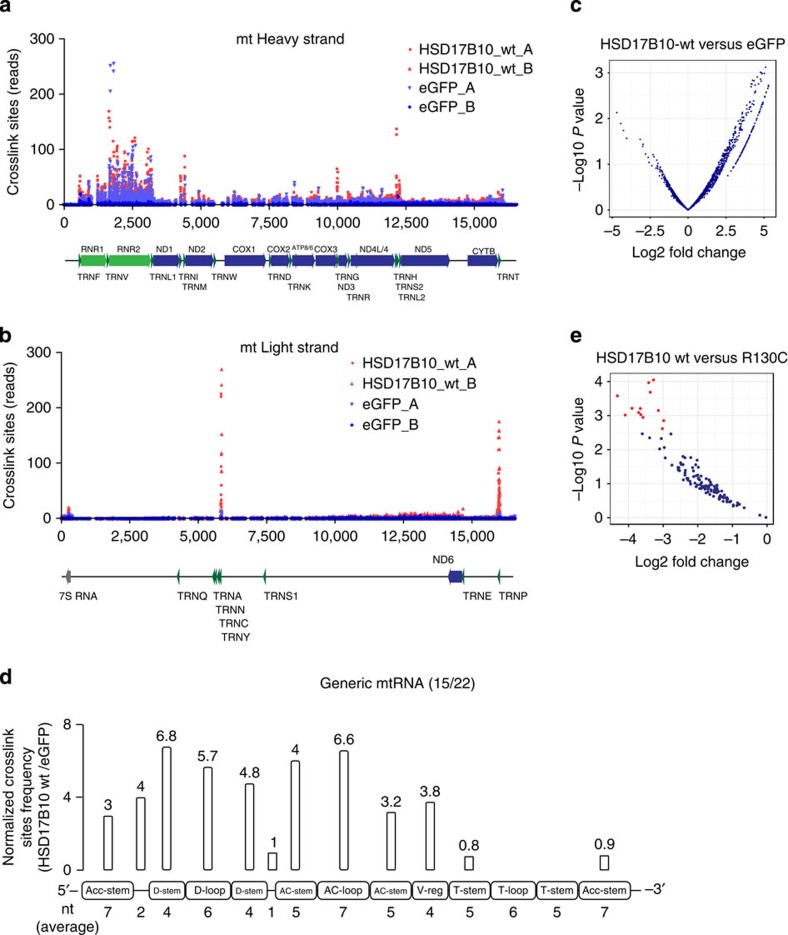
HSD17B10 iCLIP. (**a**,**b**) Raw crosslink sites reads from for HSD17B10 wt and eGFP plotted on heavy and light mt strand, respectively. (**c**) Enrichment plot for differential crosslink sites on mt RNAs for HSD17B10 wt compared with eGFP. (**d**) Average fold enrichments for the HSD17B10/eGFP of the crosslink sites plotted on generic tRNA bound by HSD17B10 (15/22), normalized by the region length. (**e**) Enrichment plot for differential crosslink clusters on mt RNAs for HSD17B10 wt compared with the R130C disease variant.

To explore the binding specificity of metabolic enzymes for RNA, we performed iCLIP^19^ and determined the interacting RNAs of the mitochondrial enzyme HSD17B10, which is mutated in patients with a mitochondrial cardiomyopathy/neuropathy syndrome (OMIM 300438), and for which a non-enzymatic function has been suspected to account for the disease phenotype^20^. HSD17B10 has been described as a subunit of the RNase P complex^21^ (together with TRMT10C and MRPP3) that processes mitochondrial tRNAs, which are interspersed within polycistronic mitochondrial transcripts^22^. Of note, MRPP3 did not appear in any of the human mRNA interactome data sets, nor could we detect ultraviolet-crosslinked RNAs on the protein (Fig. 1e). Thus, the RNA binding presumably resides on TRMT10C (RNA methyltransferase), the tetrameric HSD17B10 (dehydrogenase) or the complex of these two^23^. We observed enriched binding of HSD17B10 to mitochondrial RNAs (Supplementary Fig. 4a,b), and compared the RNA-protein crosslink sites of wt HSD17B10 to an eGFP background control (Fig. 4a–c). We found that HSD17B10 preferentially binds at the 5′ends of tRNAs (Fig. 4d), especially their D-stem, D-loop and anticodon stem and loop regions, on 15 out of 22 mt tRNAs (Supplementary Fig. 5); suggesting that the mitochondrial RNAse P does not mediate processing of all tRNAs. Indeed, processing of tRNAs that are encoded in clusters (tRNA^His^, tRNA^Ser(AGY)^, tRNA^Leu(CUN)^, Supplementary Fig. 5) was suggested to be mediated by the combination of RNAse P and the ELAC2 complex, which processes 3′ end of mt tRNAs^24^. Next, we performed iCLIP on the disease-associated variant, HSD17B10 R130C, that causes the classical phenotype of HSD10 disease^20^, retains the ability for tetramerization and displays a reduced interaction with TRMT10C *in vitro*^25^. Of note, the R130C variant exhibits a decreased binding signal to several pre-tRNAs (Fig. 4f; Supplementary Data 4). Our data identify the mitochondrial enzyme HSD17B10 as the RNA-binding subunit of RNaseP *in vivo*, and reveals that the R130C mutant is deficient in binding of a subset of pre-tRNAs. They also identify an RBP from the dehydrogenase enzyme family with clear RNA-binding specificity.

## Discussion

Taken together, the data identify a surprisingly high number of RBPs in yeast and humans, including many previously well-characterized proteins that emerge to have conserved RNA-binding activity *in vivo* (enigmRBPs). Although *in vivo* RNA binding does not prove physiological function per se, we note that the enzyme β-hydroxysteroid dehydrogenase displays remarkable RNA-binding specificity (Fig. 4). Moreover, the two enzymes aconitase 1 (ref. 18) and GAPDH^16^ are known to function as regulatory RNA-binding proteins *in vivo*, suggesting that other enigmRBPs may also moonlight as post-transcriptional regulators^5^. Alternatively, RNAs could regulate enigmRBPs: by competition with substrates for binding sites within enzymes, as allosteric regulators, or as assembly scaffolds for alignment of enzymes in a biochemical pathway^26^,^27^. RNA binding could also influence the folding, assembly or fate of newly synthesized proteins emerging from the ribosome, especially considering the fact that lysine and arginine-rich sequences have a propensity to induce ribosome stalling and protein degradation^28^,^29^. The innate immune effectors PKR, TLR3, TLR7, TLR8 and RIG-I are controlled by pathogen-derived RNAs^30^,^31^. We propose that endogenous ‘effectorRNAs' could serve roles akin to protein–protein interactors for enigmRBPs, and endow the genome with the possibility to affect mature proteins.

## Methods

### Yeast cultures

Yeast colonies (BY4741, TAP-tagged strains) were used to inoculate a 5 ml YPAD pre-culture overnight at 30 °C and 160 r.p.m. The next day, 1 litre SC-medium_120 μM Ura_ (YNB, SD-URA, 120 μM Ura, 1% glucose) cultures were started with an OD_600_ of 0.01–0.05.

### HuH-7 cell culture

Cells (kind gift from M. Muckenthaler, MMPU, Heidelberg) were cultured in low glucose (5 mM) DMEM supplemented with 10% heat inactivated FCS (PAA). For the validation procedures, we derived a HuH-7 Flp-In TREx cell line using published protocols (Flp-In T-Rex, Life), and prepared stably expressing tetracycline-inducible cell lines with genes of interest following manufacturer's instructions. The cells were grown in medium containing blasticidine (5 μg ml^−1^) and zeocin (100 μg ml^−1^) or hygromycine (200 μg ml^−1^). Transfections were done using Lipofectamine (18324-012, Life).

### Cloning

Human genes of interest were cloned into pcDNA5_FRT_TO (Life). The detailed cloning strategies and primer sequences are described in Supplementary Table 2.

### mRNA interactome capture

For HuH-7 cells, experiments were done with minor modifications in the cell lysis procedure to previously described procedure^7^. The cells were washed twice with PBS on ice before ultraviolet crosslinking. After crosslinking, the cells were lysed directly with lysis buffer on the cell culture plates, scraped and collected into 50 ml tubes. Lysates were sheared through 27G needle and incubated with oligo d(T) beads (volume ratio lysate to beads 15:1) for 1 h at 4 °C. Beads were then washed twice with each wash buffer and pooled elutions from three rounds of purifications were used for RNase treatment and subsequent processing for mass spectrometry.

For yeast mRNA interactome capture, cells were grown as described above to an OD_600_ of 0.5 before adding 4-thiouracil (4tU, Sigma 440736) to a final concentration of 500 μM. Cells were allowed to grow for 3 h before harvesting by centrifugation (4,000 r.p.m.; 15 min; 4 °C). The cell pellet was dissolved in 40 ml cold water and spread onto two Petri dishes. Ultraviolet crosslinking was performed on ice in a Spectrolinker device (Spectronics, XL1500F/A) emitting Ultraviolet light at 365 nm wavelength using energies from 0.72 to 7.2 J cm^−2^. Cells were re-pelleted (4,000 r.p.m.; 5 min; 4 °C), and pellets were dissolved in 2 ml lysis buffer (20 mM Tris pH 7.5, 500 mM LiCl, 0.5% LiDS, 1 mM EDTA, 5 mM dithiothreitol (DTT), 1 × protease inhibitor mix (EDTA-free, Roche), 1 mg ml^−1^ RNasin, 200 mM VRC). Cells were distributed into 2 ml screw-capped tubes containing an equivalent of 300 μl acid-washed glass beads, and lysed in a FastPrep device (MP bio; 6 m s^−1^; 5 × 60 s bursts with 20 s pausing in between). The lysate was cleared by centrifugation (12,000 r.p.m.; 2 min; 4 °C) and the supernatant was transferred to a 50-ml tube before snap-freezing in liquid nitrogen and storage at −80 °C. After adding lysis buffer to 25 ml, the remaining protocol was performed as described^7^ using 1 ml oligo d(T) beads per litre of starting culture. Elutions from two rounds of purification were pooled before downstream processing.

### Notes on *in vivo* labelling and crosslinking

Note that for Photoactivatable-Ribonucleoside-Enhanced Crosslinking (PAR-CL) we used 4-thiouracil (4tU) for yeast and 4-thiouridine (4SU) for HuH-7 cells, respectively. Conventional crosslinking (cCL) in yeast did not yield satisfactory results, because titration experiments using UV_254_ dosages of 0.04–1.2 J cm^−2^ revealed either insufficient RBP crosslinking, or the integrity of total RNA and RNA after oligo-d(T) selection (see above) using an RNA Pico Chip (Agilent BioAnalyzer 2100) was found to be compromised (as indicated by the decrease of the ribosomal RNA peaks) already after limited irradiation with ultraviolet light at 254 nm (Supplementary Fig. 1). This UV_254_-induced RNA damage represents the likely cause of low RBP recovery by the cCL protocol, which was hence not pursued further.

### Peptide stable isotope labelling and fractionation

Recovered proteins were digested using a combination of the endoproteinases Lys-C and trypsin, as described in detail before^3^,^4^. Protein digests were dimethyl labelled on column as previously described with slight modifications^32^. Briefly, SepPak C18 cartridges (Waters) were washed with acetonitrile (AcN) and conditioned with 0.1% (v/v) formic acid. Acidified samples were loaded and washed with 0.1% formic acid. Samples were labelled by flushing the columns with labelling reagent (using CH2O (Fisher)+NaBH3CN (Fluka) or CD2O (Isotec)+NaBH3CN). After washing with 0.1% formic acid, labelled peptides were eluted with 80% (v/v) AcN/0.05% (v/v) formic acid. Samples were mixed in a 1:1 ratio based on the total peptide amount, determined by analysing an aliquot of the labelled samples on regular LC-MS runs and comparing overall peptide signal intensities. Samples were dried by vacuum centrifugation, reconstituted in IPG rehydration buffer (8M urea, 0.4% DTT, 1% CHAPS, 2.5% Pharmalyte) and fractionated according to manufacturer's instructions using pH 3–10 IPG strips and 3100 OFFGEL fractionator (Agilent). The 12 fractions resolved were acidified and desalted with C18 Stagetips (Empore 3M)^33^. Peptide samples were dried by vacuum centrifugation and stored at −20 °C until further use.

### LC-ESI-MS/MS analysis

Peptide samples were analysed by LC-MS/MS as described in detail before^2^,^7^. In brief, peptides were separated using a nanoACQUITY UPLC system (Waters) fitted with a trapping column (nanoAcquity Symmetry C18, 5 μm, 180 μm × 20 mm) and an analytical column (nanoAcquity BEH C18, 1.7 μm, 75 μm × 200 mm). Peptides were resolved in a gradient of AcN in 0.1% (v/v) formic acid, increasing the percentage of AcN from 3 to 7% in 10 min, then to 25% in 100 min and finally to 40% in a further 10 min. Eluting peptides were analysed by direct coupling to an OrbitrapVelos Pro (Thermo Fisher Scientific) using a Proxeon nanospray source. Full scan spectra from *m*/*z* 300 to 1,700 at resolution 30,000 (profile mode) were acquired in the Orbitrap. The filling time was set at a maximum of 500 ms with limitation of 10E6 ions. The most intense ions (up to 15) carrying multiple charges (2+ and 3+) were selected for fragmentation in the ion trap. Normalized collision energy of 40% was used, and fragmentation was performed after accumulation of 3 × 10E4 ions or after filling time of 100 ms for each precursor ion (whichever occurred first). Dynamic exclusion of 30 s was applied.

### Protein identification and quantification

MS raw data files were processed with MaxQuant (version 1.2.2.5)^34^. Enzyme specificity was set to trypsin/P and a maximum of two missed cleavages were allowed. Cysteine carbamidomethylation and methionine oxidation were selected as fixed and variable modifications, respectively. The derived peak list was searched using the built-in Andromeda search engine (version 1.2.2.5) in MaxQuant against the Uniprot human database (2013_03) or *S. cerevisiae* database (2013_01), respectively, to which 248 frequently observed contaminants as well as reversed sequences of all entries had been added. Initial maximal allowed mass tolerance was set to 20 p.p.m. for peptide masses, followed by 6 p.p.m. in the main search, and 0.5 Da for fragment ion masses. The minimum peptide length was set to six amino-acid residues, and three labelled amino-acid residues were allowed. A 1% FDR was required at both the protein level and the peptide level. In addition to the FDR threshold, proteins were considered identified if they had at least one unique peptide. Peptide identifications were transferred between matching runs, based on the retention time (2 min window) and the accurate peptide masses determined in the Orbitrap analyzer. Protein identification was reported as an indistinguishable ‘protein group' if no unique peptide sequence to a single database entry was identified. Protein quantification was based on unique and razor peptides.

### Definition of mRNA interactome proteins

Peptide UniProt accession numbers were converted into ENSEMBL gene IDs. Where multiple ENSEMBL gene IDs applied, the peptide group was not considered. Statistical analysis was performed using an empirical Bayes moderated *t*-test within the Limma package in R/Bioconductor^35^. *P* values were adjusted for multiple testing using the method of Benjamini and Hochberg. Proteins within FDR 1% were considered as mRNA interactome proteins.

### SDS–PAGE, western blotting and silver staining

The procedures were performed according to the standard protocols. Antibodies used were anti-eGFP (1:3,000, 3H9, Chromotek), PUB1 (1:3,000, kind gift from Maurice Swanson), tubulin (1:3,000, ab6161, Abcam), histone H4 (1:1,000, 2592, Cell Signaling), PTBP1 (1:1,000, 5725M1, Sigma), β-actin (1:2,000, A5441, Sigma) and CSDE1 (1:2,000, 13319-1-AP, PTG). The TAP-tagged yeast proteins were visualized by staining with anti-rabbit secondary antibodies conjugated to HRP (1:10,000, NA934V, GE). Uncropped blots are shown in the Supplementary Fig. 6.

### Polynucleotide kinase assay

Cells expressing tagged genes of interest were ultraviolet crosslinked, lysed (100 mM KCL, 5 mM MgCl_2_, 10 mM Tris pH 7.5, 0.5% NP40, 1 mM DTT, protease inhibitors) and homogenized with ultrasound (3 × 10 s, 50% amplitude) on ice. Cleared lysates were treated with 50 U ml^−1^ DNAseI (Takara) and 8 ng μl^−1^ RNase A (Sigma) for 15 min at 37 °C, and used for immunoprecipitation with anti-eGFP coupled to magnetic beads (Chromotek) for 2 h at 4 °C. Beads were washed 4 × with washing buffer (500 mM NaCl, 20 mM Tris pH 7.5, 1 mM MgCl_2_, 0.05% NP40, protease inhibitors) and 2 × with polynucleotide kinase (PNK) buffer (50 mM Tris pH 7.5, 50 mM NaCl, 10 mM MgCl_2_, 0.5% NP40, 5 mM DTT). Beads were resuspended in PNK buffer with 0.1 μCi μl^−1^ [γ-32 P]rATP (Hartmann) and 1 U μl^−1^ T4 PNK (NEB) and labelled for 15 min at 37 °C. After five washes with PNK buffer without DTT, beads were boiled and loaded onto SDS–PAGE gels. The blot was then autoradiography exposed. IP efficiency was controlled by anti-eGFP antibody (1:3,000, 3H9, Chromotek). Uncropped blots and phosphorimages are shown in the Supplementary Fig. 6.

### RBPs classifications

We used the Gene Ontology (GO) database to curate the protein list (ENSEMBL gene identifiers). We assigned proteins as ‘linked mRNA biology' if their associated GO terms contained at least one of the following terms:

- ‘mRNA'
- ‘splic'
- ‘RNA binding'
- ‘RNA'
- ‘RNP'
- ‘translation'
- ‘ribosom'
- ‘nuclease'
- ‘exosome'

Some of the above terms were shortened from original words on purpose to capture multiple variations of these. If the protein did not fall in this category, we assigned it as ‘unknown' in RNA biology. Domain classification based on Interpro and Pfam domains was described in ref. 2. We devised new category for RBD domains ‘recognized' that encompasses both ‘classical' and ‘non-classical' categories from ref. 2.

### Complete human mRNA interactome data set

For the complete human mRNA interactome data set, we used the combination of proteins found in this study (FDR 1%), proteins from HeLa mRNA interactome^2^ and HEK293 cell RNA-binding proteins (Supplementary Data 1)^3^.

### Complete yeast mRNA interactome data set

For the complete yeast mRNA interactome data set, we combined the proteins of this study (FDR 1%) and the proteins from ref. 9.

### Disordered regions and low complexity

The intrinsic disorder of proteins is computed with IUPred^36^. Disordered amino-acid residues are defined by a IUPred score of >0.4. For each protein, the fraction of disordered amino-acid residues is computed. To assess complexity, shannon entropy is computed for each amino acid position within a window of ±10 residues. Positions with an entropy <3 bits are considered as low complexity. For each protein, the fraction of amino-acid residues in low-complexity regions is computed.

### Ortholog definition

To define orthologs of yeast and human, ENSEMBL gene ID were converted to UniProt IDs and used for the InParanoid ortholog groups database (Release 7.0, June 2009)^37^. There were 2,041 InParanoid ortholog groups in total, covering 3,670 human and 2,386 yeast proteins. InParanoid clusters were categorized according to their mRNA-binding behaviour. An InParanoid cluster was regarded as mRNA binding, if at least one of the contained proteins is included in the complete human or yeast mRNA interactome data set. This categorization resulted in three groups of InParanoid clusters: some showed mRNA binding in human and yeast, some showed mRNA binding only in human and some did not show mRNA binding either in yeast or in human. There was not a single InParanoid cluster that shows mRNA binding in yeast, but not in human.

### K-mers motif evolution across species

The InParanoid database was used to find clusters from *Caenorhabditis elegans* (*C. elegans*), *Drosophila melanogaster* (*D. melanogaster*) and *Danio rerio* (*D. rerio*), which contain orthologs to the 2,041 ortholog groups described above. Within each InParanoid cluster, the protein with the longest (isoform) sequence was chosen as the representative for the cluster. For each yeast–human orthology group, one orthologous protein in fish (respectively, fly and worm) was selected. Selection was based on orthology to the representative protein in yeast and the representative protein in human. If there were multiple proteins that fulfilled the previous condition, the protein with the largest number of amino acids was chosen. A list of K-mers was computed for each organism, providing a vector of K-mers for each protein in each organism. Next, we created a list of all K-mers appearing in the conserved human and yeast proteins; K-mers containing an ‘X' or ‘U' were excluded. A table counting the repeat number of each motif in each protein was computed for each of the five organisms. The tables were combined to a three-dimensional array (proteins × motifs × organisms). For each motif *n*, we tested if the mRNA-binding proteins were enriched for proteins in which the copy number of motif *n* in human was at least increased by two compared with yeast. The *P* values were computed by Fisher's exact test and *P* values were corrected for multiple testing by the method of Benjamini–Hochberg. Motifs at a FDR of 0.2 were selected as ‘increase in repeat numbers' motifs.

### GO enrichments analysis

For GO enrichment analysis, the DAVID database (version 6.7)^38^,^39^ was used. As a background, the total human or yeast proteomes, respectively, were used.

### ‘Classic' metabolic enzymes

We used the IntEnz database (http://www.ebi.ac.uk/intenz/) to classify all enzymes in the mRNA interactomes. For the purpose of this study and to exclude obvious RNA-related enzymes, we did not consider the following as classic metabolic enzymes:

- RNA/DNA helicases
- tRNA, rRNA modification enzymes (tRNA methyltransferase, pseudouridylases and so on)
- nucleases (RNA or DNA)
- tRNA aminoacylsynthetases
- RNA/DNA polymerases
- topoisomerases
- proteasome subunits
- regulatory subunits (of any enzyme)

### iCLIP and data analysis

iCLIP was performed with following modifications. Stably expressing HuH-7 cells were induced overnight with 100 ng ml^−1^ of tetracycline, ultraviolet-crosslinked and lysed on plate. Lysates were homogenized using Branson sonifier (3 × 10 s, 50% amplitude) and cleared at 13,000 r.p.m. for 10 min. IP with anti-GPF magnetic beads was performed as described above and following washes were applied twice each: high-salt wash (500 mM NaCl; 20 mM Tris pH 7.5; 1 mM MgCl_2_; 0.05% NP40; 0.1% SDS), medium-salt wash (250 mM NaCl; 20 mM Tris pH 7.5; 1 mM MgCl_2_; 0.05% NP40) and low-salt wash (150 mM NaCl; 20 mM Tris pH 7.5; 1 mM MgCl_2_; 0.01% NP40). After RNase treatment and dephosphorylation of 3′ ends, RNA linker was ligated overnight at 850 r.p.m. at 16 °C. Beads were treated with proteinase K and eluates were used for RNA isolation, cDNA production and sequencing following published protocol^19^. Low-FDR crosslink sites read values were then used for the DESeq analysis^40^.

## Additional information

**How to cite this article**: Beckmann, B. M. *et al*. The RNA-binding proteomes from yeast to man harbour conserved enigmRBPs. *Nat. Commun.* 6:10127 doi: 10.1038/ncomms10127 (2015).

## Supplementary Material

Supplementary Figures and Tables Supplementary Figures 1-6 and Supplementary Tables 1-2

Supplementary Data Set 1 Yeast Interactomes

Supplementary Data Set 2 Human Interactomes

Supplementary Data Set 3 Wt-vs-R130C
