# Supplementary material for: The RNA-binding proteomes from yeast to man harbour conserved enigmRBPs
Source: Nat Commun. 2015 Dec 3;6:10127. doi: 10.1038/ncomms10127 (PMC4686815; doi:10.1038/ncomms10127)
Supplement: Supplementary Figures and Tables — Supplementary Figures 1-6 and Supplementary Tables 1-2 [file ncomms10127-s1.pdf]

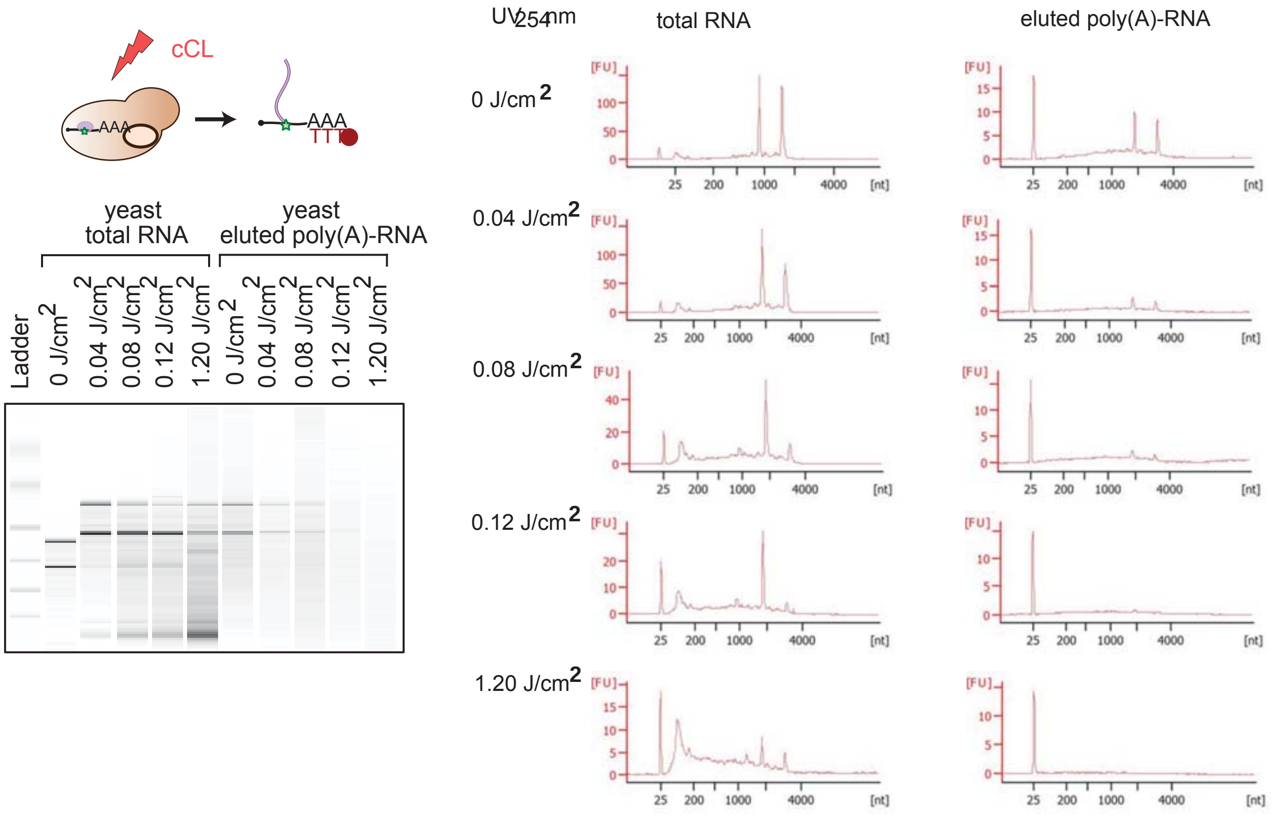

Supplementary Figure 1: UV_{254}-induced RNA damage in S. cerevisiae

Analysis of yeast RNA integrity after 254 nm crosslinking (cCL): After irradiation of living yeast cells with 0 – 1.2 J/cm^2 of UV_{254}, RNA integrity was analyzed using a BioAnalyzer 2100 (RNA Pico Chip). The top panel displays the gel representation of in-chip electrophoresis , the bottom panel the respective diagrams including the two ribosomal RNA peaks.

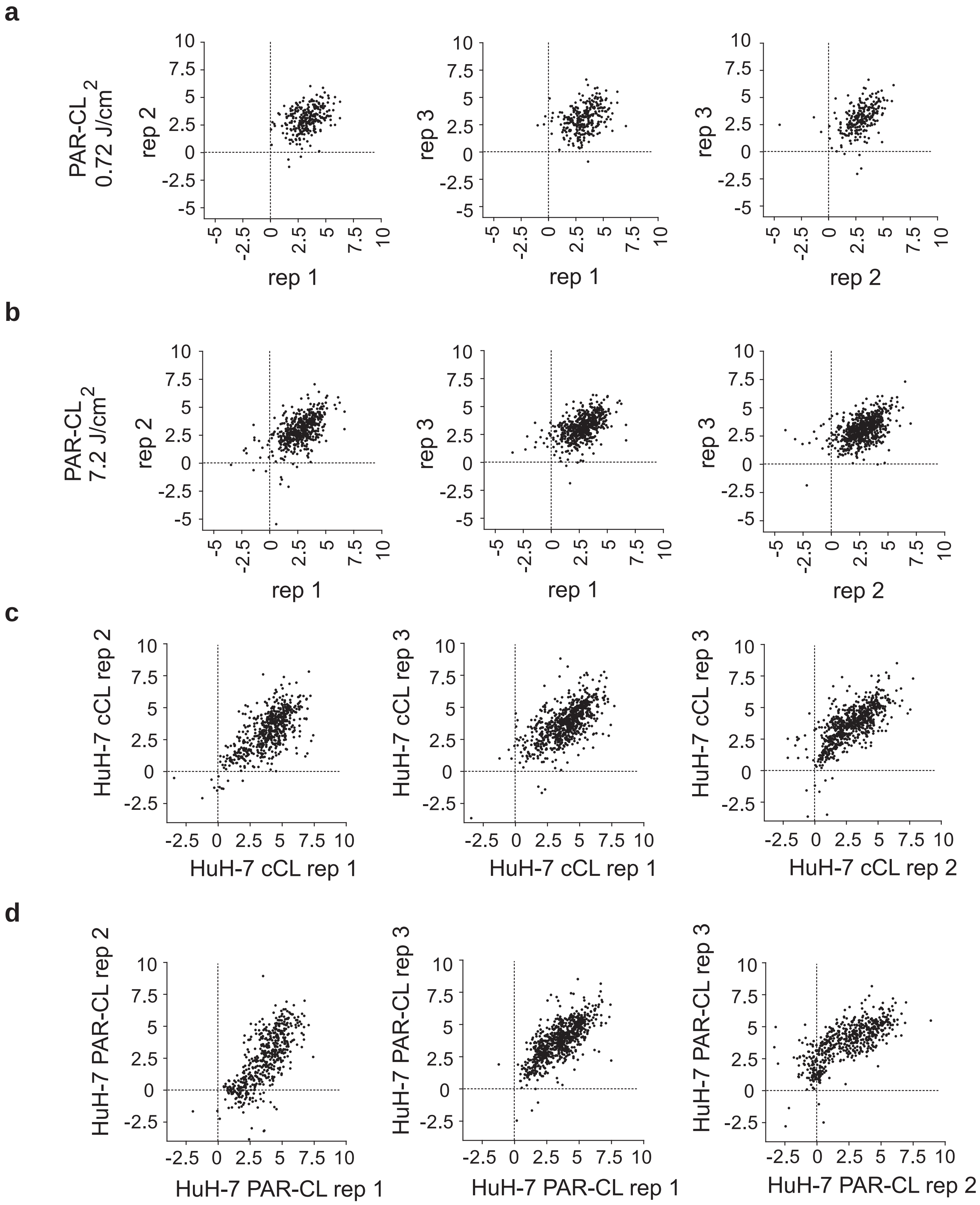

Supplementary Figure 2: Replicate mRNA interactome experiments

Three biological replicates of mRNA interactome capture from yeast (a, b) and HuH-7 (c, d) are plotted against each other based on the protein enrichment in crosslinked over non-crosslinked samples.

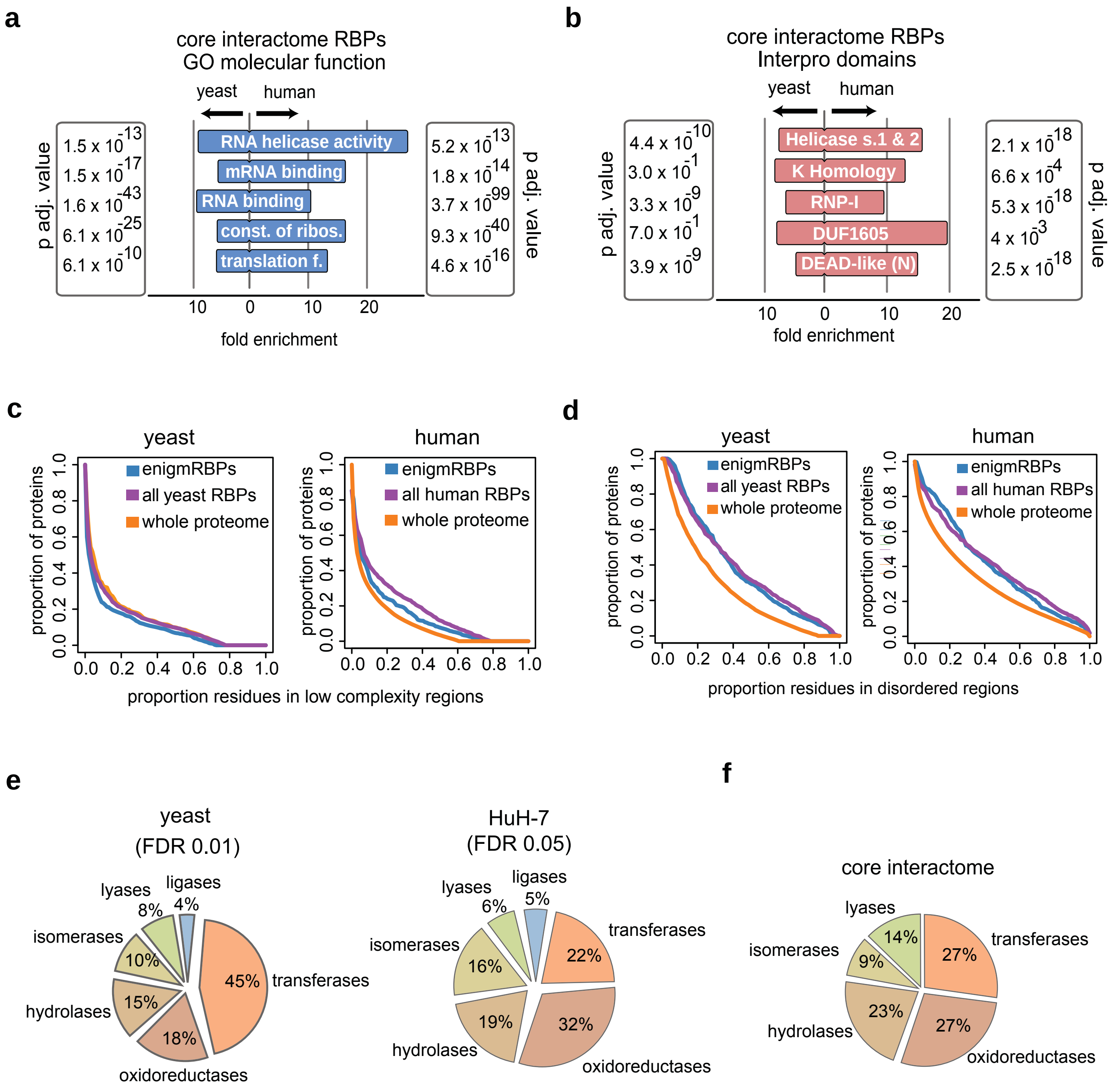

Supplementary Figure 3: Characteristics of yeast and HuH-7 mRNA interactomes

a, GO biochemical pathway enrichment of the core mRNA interactome. b, Protein domain enrichment of the core mRNA interactome. c, Proportions of the amino acids in low complexity regions of enigmRBPs, all interactome RBPs and whole proteomes of yeast and human. d, Proportions of the amino acids in disordered regions of enigmRBPs, all interactome RBPs and whole proteomes of yeast and human. e, EC enzyme classification of enzyme RBPs from yeast and HuH-7 cells. f, EC enzyme classification of enzyme RBPs from core mRNA interactome.

a

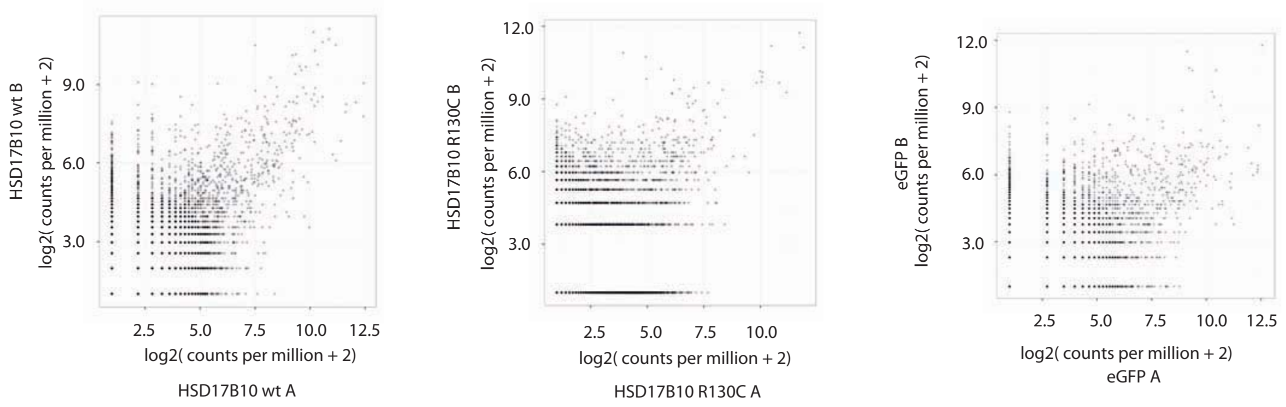

b

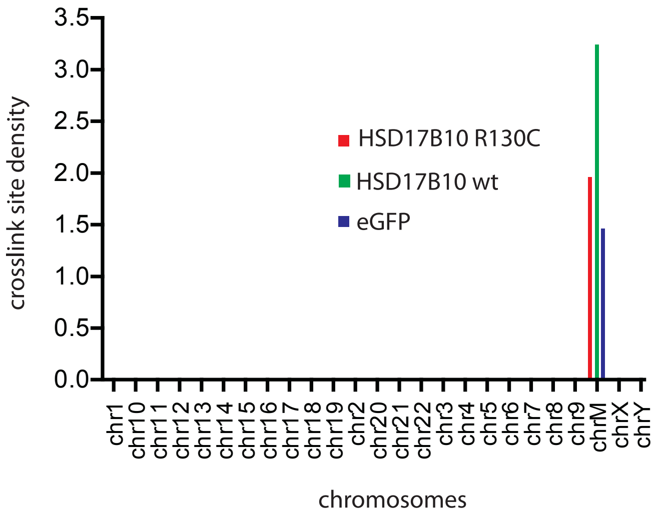

Supplementary Figure 4: HSD17B10 iCLIP

a, iCLIP replicates correlation of bound RNA species. Displayed are crosslink peaks as log2 (counts per million + 2). b, Crosslink site bar plot for iCLIP cDNA libraries after alignment to the human genome and random barcode evaluation.

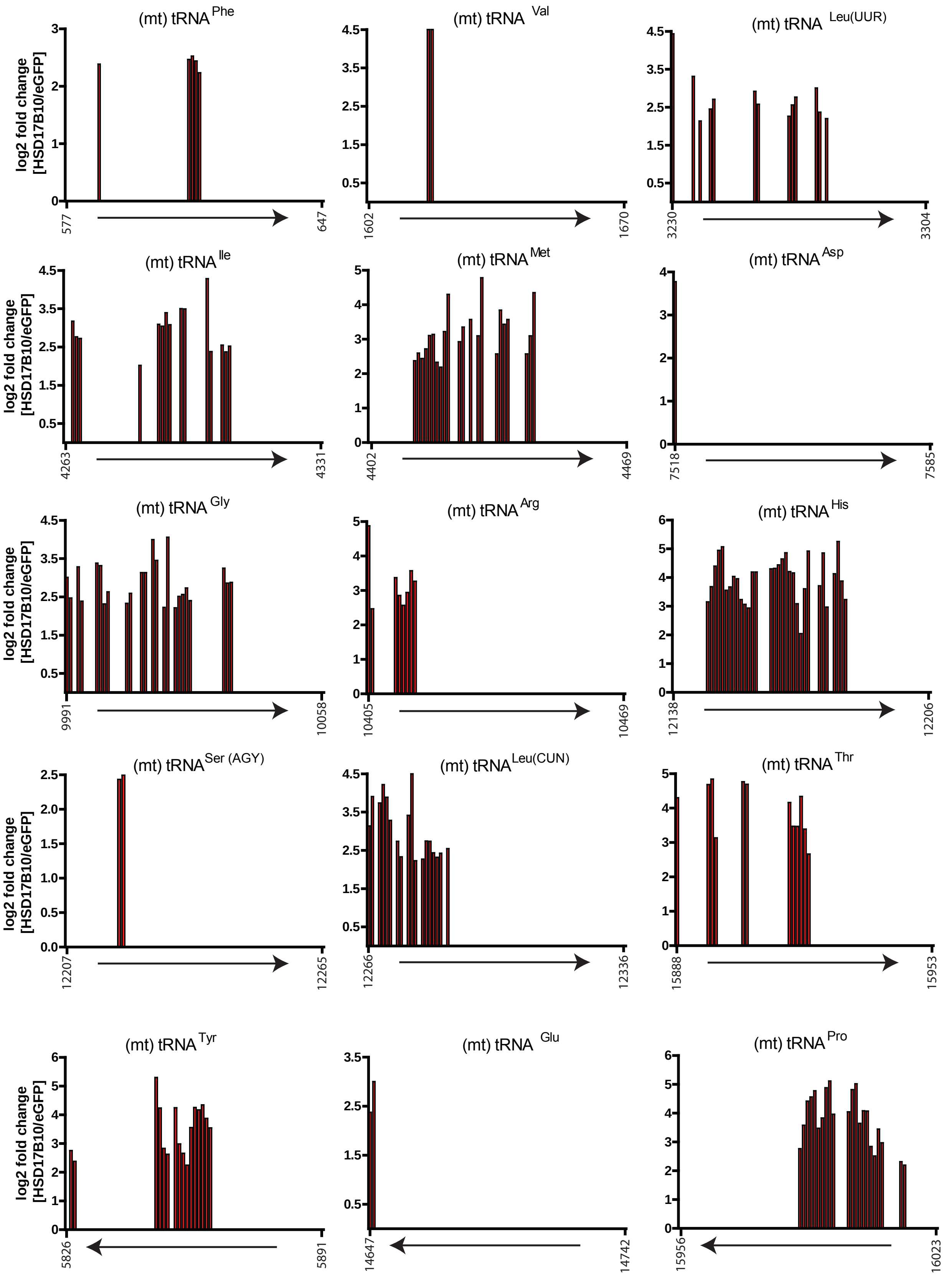

Supplementary Figure 5: HSD17B10 bound mt tRNAs

Displayed are mt tRNAs bound by HSD17B10, with each bar representing log2 fold enrichment of crosslinked nucleotide site over eGFP control ($p<0.1$). X axis represents nucleotide positions, arrow indicates transcriptional direction.

Uncropped blots related to Figure 1a

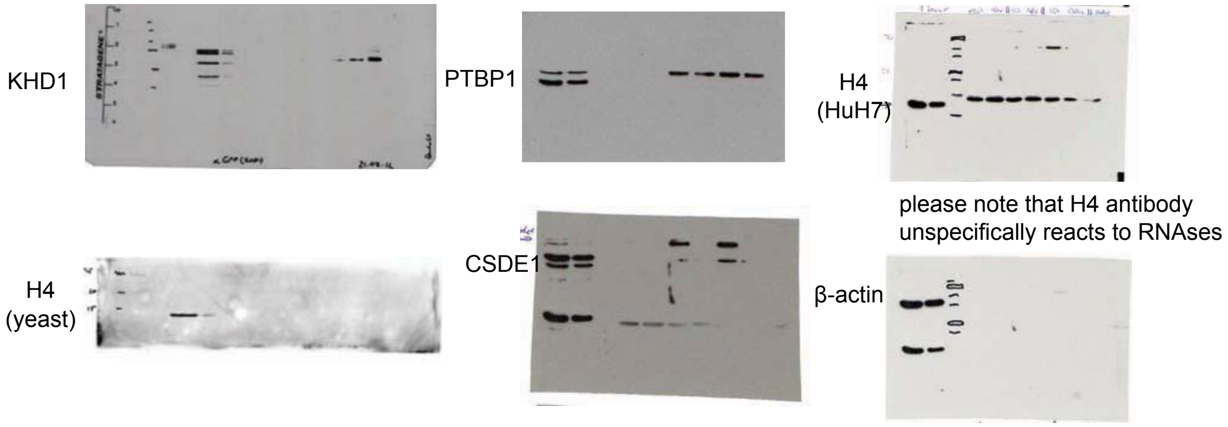

Uncropped blots related to Figure 1d

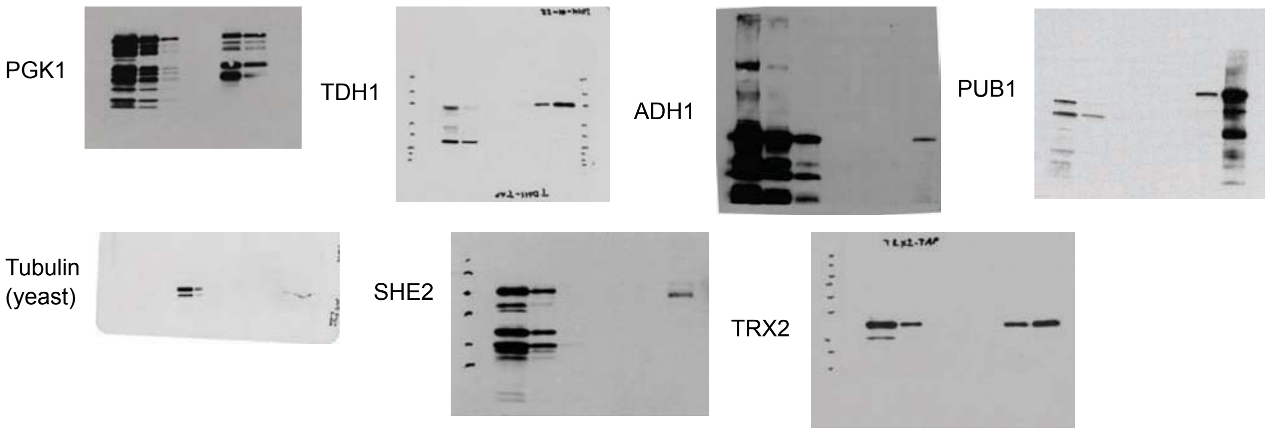

Uncropped blots related to Figure 1e

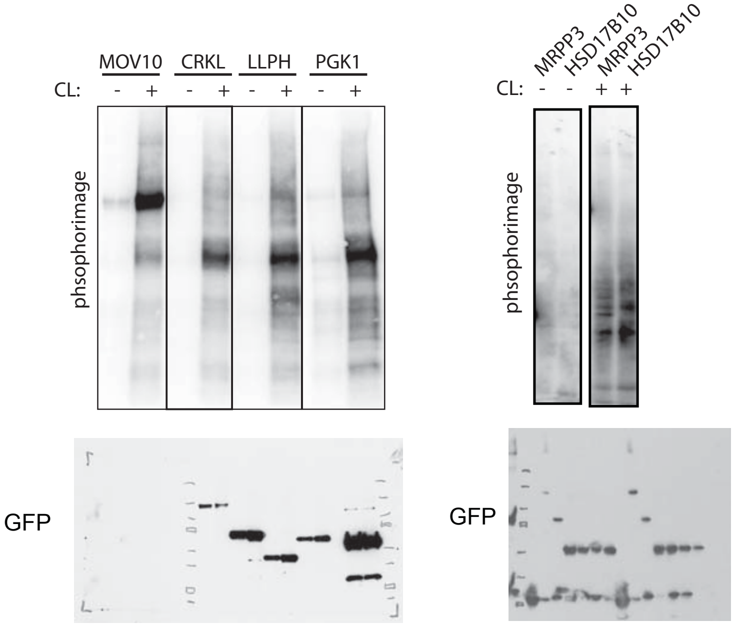

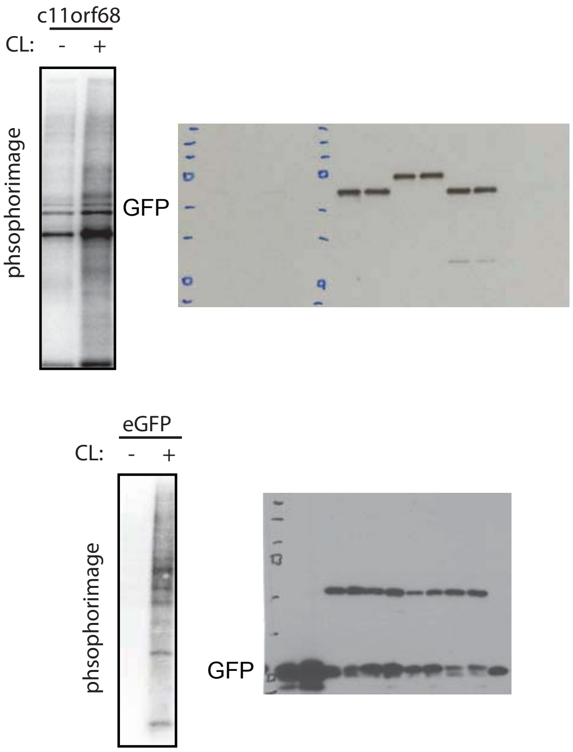

Supplementary Figure 6: Uncropped blots and phosphorimages related to Figure 1

Supplementary Table 1. Examples of enigmRBPs

**Function**	**# Yeast RBPs**	**Examples Yeast**	**# Human RBPs**	**Examples Human**
Glycolysis	17	**CDC19, PGI1, GLK1, PGK1, ENO1, ENO2, FBA1**, GPM1, **TDH1, TDH3**, HXK1, HXK2, EMI2, PFK1, PFK2, **TPI1**, ADH1	9	**PGK1, GAPDH,** LDHA, **TPI1, ALDOA, ALDOC, PKM, ENO1, GPI**
Thioredoxin system	8	TRR1, AHP1, **TSA1**, GRX1, GRX2, **TRX1**, **TRX2**, YFR016C	10	**PRDX1**, PRDX2, **PRDX3**, **TXN**, PDIA3, PDIA4, P4HB, DYNC1H1, TXNDC5, GLRX3
Actin-binding	5	ARP3, ARC40, AIP1, SAC6, ABP1	41	EZR, FLNB, TLN1, RDX, FLNA, ANXA2, TWF2, SPTAN1, …
Protein-folding	13	BUD27, **CCT4**, CPR1, CPR6, **TCP1**, ZUO1, …	32	FKBP4, **CCT4**, PPIA, BAG4, CANX, **TCP1**, …
Protein kinases	21	FUS3, YPK1, CTK1, YCK2, KIN2, CMK1, …	21	MARK2, CDK13, PKN2,SRPK1, TRIM28, …
Ubiquitination	6	UBA1, UBC1, UPF3, NAM7, **CDC48**, ...	15	UBA1, DCAF13, **VCP**, RC3H1, …

proteins in bold letters are orthologous RBPs in yeast and human

Supplementary table 2

Cloning of human ORFs of interest for validation. eGFP only and the MOV10-YFP construct were described[2]. For HSD17B10, MRPP3 (KIAA0391) the cDNA was PCR-fused to eGFP, therefore we list two primers for 3' restriction endonuclease. Genes were amplified from HuH-7 cDNA using primers with restriction endonuclease sites as follows:

Gene	eGFP position on	Linker AA sequence	F-RE	R-RE
C11orf68	N-term	GGSGGSGGGTELGS	BamHI 5'-AAGGATCCGAACCAGGTGAGGAGCTGG-3'	XhoI 5' AACTCGAGTTACTAGGTCAGTTCCACGTTG-3'
CRKL	C-term	LEGGSGGSGG	KpnI 5'-AAGGTACCATGTCCTCCGCCAGGTTCGAC-3'	XhoI 5'-AACTCGAGCTCGTTTTCATCTGGGTTTTGAG-3'
HSD17B10	C-term	none	HindIII 5'-AAAAGCTTACCATGGCAGCAGCGTGTCGGAGC-3'	XhoI 5'-GGATGGGGCCATTCGTATGCAGCCTGTGAGCAAGGGCGAGGAGCTGTTCACC-3' and 5'-GGTGAACAGCTCCTCGCCCTTGCTCACAGGCTGCATACGAATGGCCCCATCC-3'
LLPH	C-term	LEGGSGGSGG	BamHI 5'-AAGGATCCACCATGGCTAAAAGCTTACGGAGT-3'	XhoI 5'-AACTCGAGCCAGGCCAAACCCTTTGCCAC-3'
MRPP3	C-term	GGSGGSGG	BamHI 5'-AAGGATCCACCATGACTTTCTATTTGTTTGGTATTCG-3'	XhoI 5'-GCTTTGCCTCCACCAAAAGACAGGAGGTTCAGGTGGATCTGGAGGTGTGAGCAAGGGCGAGGAGCTGTTCACC-3' 5'-GGTGAACAGCTCCTCGCCCTTGCTCACACCTCCAGATCCACCTGAACCTCCTGTCTTTTGGTGGAGGCAAAGC-3'
